# Diagnostic accuracy of cardiovascular magnetic resonance imaging of right ventricular morphology and function in the assessment of suspected pulmonary hypertension results from the ASPIRE registry

**DOI:** 10.1186/1532-429X-14-40

**Published:** 2012-06-21

**Authors:** Andrew J Swift, Smitha Rajaram, Robin Condliffe, Dave Capener, Judith Hurdman, Charlie A Elliot, Jim M Wild, David G Kiely

**Affiliations:** 1 National Institute of Health Research, Cardiovascular Biomedical Research Unit, Sheffield, UK; 2 Unit of Academic Radiology, University of Sheffield, Sheffield, UK; 3 Sheffield Pulmonary Vascular Disease Unit, Royal Hallamshire Hospital, Sheffield Teaching Hospitals NHS FoundationTrust, Sheffield, UK; 4 University of Sheffield, Academic Unit of Radiology, C Floor Royal Hallamshire Hospital, Glossop Road, Sheffield, S10 2J, UK

**Keywords:** Pulmonary hypertension, Cardiovascular magnetic resonance, Ventricular mass index, Late gadolinium enhancement, Left heart disease, Pulmonary arterial hypertension, Right ventricle

## Abstract

**Background:**

Cardiovascular Magnetic Resonance (CMR) imaging is accurate and reproducible for the assessment of right ventricular (RV) morphology and function. However, the diagnostic accuracy of CMR derived RV measurements for the detection of pulmonary hypertension (PH) in the assessment of patients with suspected PH in the clinic setting is not well described.

**Methods:**

We retrospectively studied 233 consecutive treatment naïve patients with suspected PH including 39 patients with no PH who underwent CMR and right heart catheterisation (RHC) within 48hours. The diagnostic accuracy of multiple CMR measurements for the detection of mPAP ≥ 25 mmHg was assessed using Fisher’s exact test and receiver operating characteristic (ROC) analysis.

**Results:**

Ventricular mass index (VMI) was the CMR measurement with the strongest correlation with mPAP (r = 0.78) and the highest diagnostic accuracy for the detection of PH (area under the ROC curve of 0.91) compared to an ROC of 0.88 for echocardiography calculated mPAP. Late gadolinium enhancement, VMI ≥ 0.4, retrograde flow ≥ 0.3 L/min/m^2^ and PA relative area change ≤ 15% predicted the presence of PH with a high degree of diagnostic certainty with a positive predictive value of 98%, 97%, 95% and 94% respectively. No single CMR parameter could confidently exclude the presence of PH.

**Conclusion:**

CMR is a useful alternative to echocardiography in the evaluation of suspected PH. This study supports a role for the routine measurement of ventricular mass index, late gadolinium enhancement and the use of phase contrast imaging in addition to right heart functional indices in patients undergoing diagnostic CMR evaluation for suspected pulmonary hypertension.

## Background

Pulmonary Hypertension (PH) is defined as a mean pulmonary artery pressure (mPAP) greater than or equal to 25 mmHg measured at cardiac catheterisation [1]. It ranges from a rare, progressive condition characterised by a vasculopathy, pulmonary arterial hypertension (PAH), to mild elevations of pulmonary artery pressure commonly seen in association with respiratory and cardiac disease. In patients with PAH an increase in mPAP and pulmonary vascular resistance (PVR) results in right ventricular failure and death with a median survival in untreated patients of less than 3 years [2,3].

Right heart catheterisation (RHC) is the gold standard test used to assess for the presence or absence of PH by directly measuring mPAP and allows measurement of cardiac output (CO) and index (CI), right atrial pressure, mixed venous oxygen saturation and PVR, which are used as markers of disease severity. However, although safe in expert hands it is an invasive test and its role is limited to confirming a diagnosis of PH and in selected cases assessing the response to treatment. Echocardiography is currently the most commonly used non-invasive test in patients with suspected PH, however, this technique has a number of limitations and does not perform well for certain aetiologies of PH [4,5], significantly over and underestimating invasively measured mPAP [6]. Consequently, there is increasing interest in developing other non-invasive imaging tools.

Several studies have shown significant relationships between cardiovascular magnetic resonance (CMR) derived measurements with mPAP and PVR in patients with PH; including ventricular mass index (VMI) [7-11], inter-ventricular septal configuration [12-14], pulmonary arterial blood flow quantification and pulsatility [10,15-18]. However, these studies have primarily been limited to patients with PAH and the diagnostic utility of CMR measurements in a large population of unselected patients with PH has not been evaluated.

The aim of this study was to compare the diagnostic accuracy of a variety of CMR parameters to identify PH confirmed at cardiac catheterisation in unselected patients with suspected PH attending a referral centre.

## Methods

### Patients

Consecutive treatment naive patients undergoing RHC and CMR for suspected PH were identified between January 2008 and March 2010 at a high volume nationally designated PH referral centre. All incident patients with suspected PH routinely undergo CMR as part of their diagnostic work-up. Inclusion criteria required the patients’ RHC and CMR to be within 48 h, and patients were excluded if the imaging was of non-diagnostic quality. Other exclusion criteria were as per standard criteria for patients undergoing CMR. Approval for retrospective analysis of imaging techniques was granted by the local research ethics committee.

### CMR acquisition

CMR was performed on a 1.5 T whole body scanner GE HDx (GE Healthcare, Milwaukee, USA), using an 8 channel cardiac coil. 4 chamber and short axis cine images were acquired using a cardiac gated multi-slice balanced steady state free precession sequence (20 frames per cardiac cycle, slice thickness 8 mm, FOV 48, matrix 256 × 256, BW 125 KHz/pixel, TR/TE 3.7/1.6 ms). A stack of images in the short axis plane with slice thickness of 8 mm (2 mm inter-slice gap) were acquired fully covering both ventricles from base to apex. For short axis imaging end-systole was considered to be the smallest cavity area. End-systole was defined as maximal RV shortening on the 4 chamber slice images. End-diastole was defined as the first cine phase of the R-wave triggered acquisition for both short axis and four chamber imaging. Ten minutes following gadolinium contrast injection (0.1 mmol/kg of gadolinium-DTPA; Gadovist, Bayer, Germany), late-enhancement imaging was performed using a 3D-gradient spoiled echo sequence (repetition time 7.7 ms, echo time 3.6 ms, TI 180 ms, slice thickness 8 mm, FOV 45 × 40.5,matrix 256 × 224). Selective 180° inversion-recovery images were acquired in the short axis obtaining limited 3–5 slices through the ventricles. We have corrected our MR parameters where appropriate for body surface area, as previously reported in the literature [19]. Phase contrast imaging parameters were as follows: repetition time 5.6 ms, echo time 2.7 ms, slice thickness 10 mm, FOV 48 × 28.8, band-width 62.5 kHz, matrix = 256 × 128, 20 reconstructed cardiac phases and velocity encoding 150 cm/s in the slice direction. Phase contrast imaging was performed orthogonal to the pulmonary arterial (PA) trunk Studies were performed with patients in the supine position with a surface coil and with retrospective ECG gating.

### Image analysis

Image analysis was performed on a GE Advantage Workstation 4.1 by a pulmonary vascular radiologist (AS) (with 2 years specialist experience in CMR) who was blinded to the patient clinical information, and cardiac catheter parameters. Patient’s scans were defined as non-diagnostic when image quality significantly affected cardiac measurements or volumetric analysis could not be accurately performed.

#### RV ejection fraction (RVEF), end-diastolic volume (RVEDVI), stroke volume (RVSVI)

**Figure 1:**
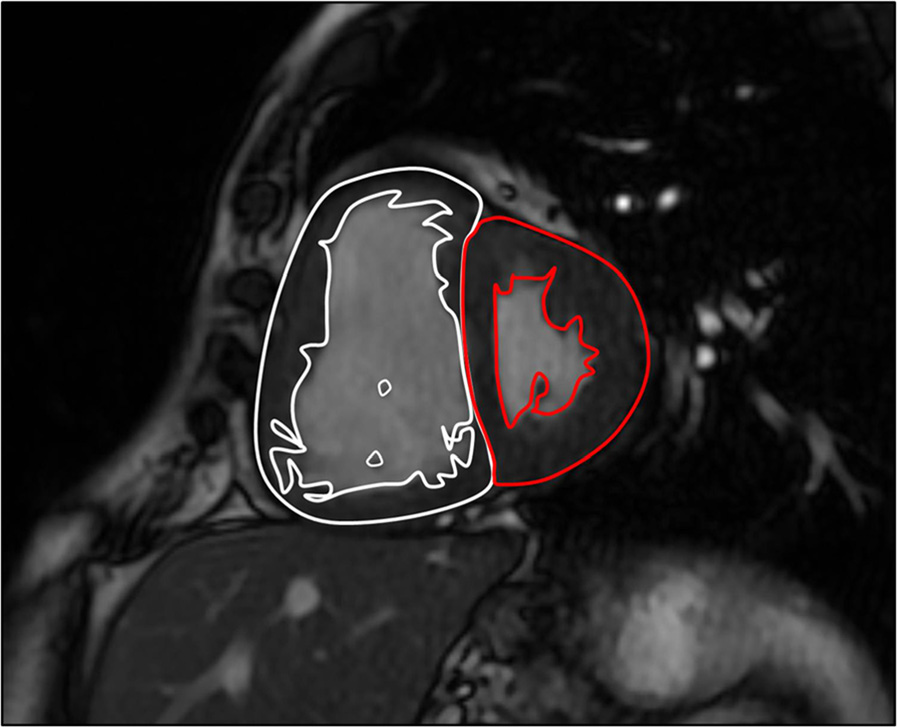
**Short axis slice through the ventricles showing epicardial and endocardial border segmentation of the RV (white) and the LV (red).** The inter-venticular septum is included in the LV mass, Ventricular mass index (VMI) is defined as RV mass divided by LV mass. The endocardial borders of the RV and LV were traced for calculation of end-diastolic volume (EDV) and end-systolic volume (ESV). EDV and ESV were calculated by summation of the product (area × slice distance) for all slices. SV is given by SV = EDV-ESV calculated for both RV and LV.

Endocardial surfaces were manually traced from the stack of short-axis cine images, using our MR workstation software (GE Advantage Workstation ReportCard) to obtain RV end-diastolic and end-systolic volumes. From end-diastolic volume and end-systolic volumes, the RVEF and RVSV were calculated. RVSVI was defined as RVSV/body surface area (BSA) measured in ml/m^2^, see Figure 1.

#### RV mass index and ventricular mass index (VMI)

The RV epicardial and endocardial borders on each end-diastolic short axis slice image were outlined. The IVS was considered as part of the LV. The myocardial volume for each slice was calculated by multiplying the area of the RV wall by the slice thickness. The product of the sum total of the myocardial slice volumes for each ventricle and the density of myocardium (1.05 g/cm3) gave an estimate of RV mass[9]. RV mass index was defined as RV mass/body surface area (BSA) measured in g/m^2^. The LV epicardial and endocardial borders on each end-diastolic short axis slice were outlined, LV end diastolic mass was thus derived. VMI was defined as RV mass divided by LV mass, Figure 1.

#### RV relative area change (RVRAC)

The endocardial contours at end-diastole and end-systole were manually segmented. RVRAC expressed as a percentage was calculated from the 4 chamber plane images using the following formula: RAC = 100[(Diastolic area-Systolic area)/Diastolic area].

#### Longitudinal function

Longitudinal RV motion was assessed using Tricuspid Annular Plane Systolic Excursion (TAPSE), a measurement previously evaluated using echocardiography and MRI [20-22]. TAPSE was calculated manually from the change of the tricuspid annulus-apex distance between end-diastole and end-systole 4-chamber images. Fractional measure of longitudinal motion utilised the tricuspid annulus apex distance change (*fractional-*TAAD) and was calculated as TAPSE divided by the tricuspid annulus-apex dimension at end-diastole expressed as a percentage. This method was previously described by Kind et al. [22].

#### Transverse function

Transverse RV function was determined from the change of the septum-free-wall perpendicular distance (SFD) at the mid-point between the apex and the base. This was measured manually as the change between the SFD at end-diastole and SFD at end-systole on the 4-chamber images. Fractional-SFD was calculated as for fractional longitudinal function [22].

#### Left ventricular eccentricity

Left ventricular eccentricity measurements have been traditionally expressed as the ratio of the length of two LV perpendicular minor-axis diameters [23]. Systolic eccentricity index measurements were obtained from the mid-chamber SA end-systolic image, the ratio was calculated by the formula sEI = D2/D1, where D2 is the diameter parallel and D1 is perpendicular to the IVS. Abnormal values derived from echocardiography are considered to be > 1.2 [23]. dEI was calculated from the mid-chamber SA end-diastolic image using methods as described.

#### Phase contrast indices – PA (n = 106)

Phase contrast CMR was performed at our institution beginning May 2009. Phase contrast Q flow CMR was analysed using Reportcard software. The following indices were generated: average velocity (cm/s), retrograde flow (L/min) and percentage retrograde flow (%) Maximal and minimal pulmonary artery areas were recorded, and relative area change (RAC) was defined by the following: (maximal area-minimum area)/minimum area.

#### Late gadolinium enhancement CMR

Late enhancement images were qualitatively assessed for hyper-intensity at the inter-ventricular septal hinge points or along the septum as previously described. The presence or absence of delayed enhancement was recorded.

#### Echocardiography

Echocardiography was performed using Powervision 6000 and 8000 machines (Toshiba, Japan) by trained cardiac physiologists as part of routine clinical care at a high volume PH referral centre using a standard protocol for these patients. Multiple windows were used to obtain the optimal Doppler estimation of tricuspid regurgitant jet velocity (TRJV) and tricuspid gradient (TG) calculated using the modified Bernoulli equation, TG = 4 x TRJV^2^ . Saline agitation was not used. Right atrial pressure was estimated from the diameter and respiratory variation of the inferior vena cava. Estimates of right atrial pressure and TRJV were used to estimate PA systolic pressure (PA systolic pressure = tricuspid gradient + estimated right atrial pressure). For the purpose of comparison with right heart catheter measures of MPAP and CMRI estimates, MPAP was defined as 0.61 x PA systolic pressure + 2 mmHg [6].

#### RHC and clinical evaluation

RHC was performed using a balloon-tipped 7.5 Fr thermodilution catheter (Becton-Dickinson, USA). Patients referred for the investigation of suspected PH also underwent clinical evaluation including; blood testing, echocardiography, computed tomography scanning, lung function testing, exercise testing and perfusion lung imaging. Diagnostic classification of the form of PH was by standard criteria following multidisciplinary assessment [24].

#### Statistics

Comparisons of CMR measurements between ‘No PH’ and PH patients were analysed using the independent *t*-test for continuous data, the chi-square for categorical data and anova testing with bonferroni corrections for multiple variables. Pearson’s correlation coefficient was used to assess the correlations between CMR parameters and RHC values. Receiver operating characteristic (ROC) analysis was used to test the diagnostic strength of CMR parameters for the detection of the presence or absence of PH. ROC curve analysis results are presented as area under the curve (AUC). The sensitivity, specificity, negative and positive predictive values of CMR indices for determining the presence or absence of PH was assessed using Fisher’s exact test. A p-value < 0.05 was considered statistically significant. To perform and display the statistics, SPSS 18 (SPSS, Chicago, Ill) and GraphPad Prism 5.03 (GraphPad Software, San Diego, Calif) software were used.

## Results

**Figure 2:**
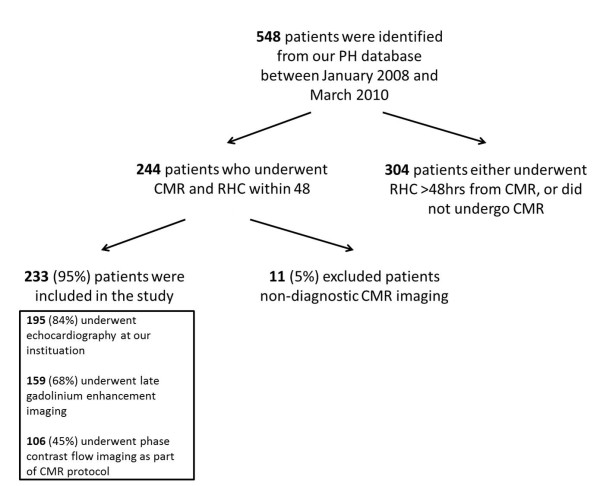
Flow chart of study profile.

548 incident, treatment naïve patients who had undergone investigation for suspected PH with a right heart catheter based approach were identified from our PH database between January 2008 and March 2010. 244 patients underwent CMR and RHC within 48 h of each other. 11 patients (5%) were excluded as their CMR scans were degraded by artefact and hence cardiac measurements and volumetric analysis could not be reliably performed in these patients. Of the remaining 233 patients, 194 had PH as defined by mPAP ≥ 25 mmHg, the remaining 39 were labelled as ‘no PH’. Patients with PH were divided into subgroups according to the Dana Point classifications, see Table 1[24]. 106 patients underwent CMR including phase contrast imaging and RHC between May 2009 and March 2010, and 159 patients underwent late gadolinium enhancement imaging. Echocardiography was performed in 195 of 233 patients at our institution. Scans in the remaining patients were performed at other institutions prior to referral and were excluded from analysis. Echocardiography was in most cases performed prior to RHC with a mean interval 35 ± 50 days. A flow chart of the study profile is shown in Figure 2.

**Table 1:** Patient Subgroup Classification

**Clinical classification of PH patients as per Dana Point[**[24]**]**	
Gp1 PAH (n = 83)	
Idiopathic	28
PAH Connective tissue diseases	39
PAH Portal hypertension	5
PAH Congenital heart disease	11
Gp2 PH due to left heart disease (n = 21)	
LV Diastolic dysfunction	21
Gp3 PH due to lung diseases and/or hypoxia (n = 29)	
Chronic obstructive pulmonary disease	19
Interstitial lung disease	5
Mixed obstructive and restrictive	7
Gp4 CTEPH	59

### Group and subgroup comparisons

Demographic information and RHC data are shown in Table 2. Patient age, male to female ratio and body surface area (BSA) were not significantly different between the PH and ‘No PH’ groups. Significantly higher values of RVEDVI, RV mass, sEI and dEI were demonstrated in the PH group, and significantly lower values of TAPSE, f-TAAD, SFD, f-SFD, RVEF, RVSVI and RVRAC were found in the PH group as compared to the ‘No PH’ group. Derived from phase contrast imaging, PA retrograde flow, systolic area and diastolic area were significantly higher in patients with PH, whereas PA average velocity and relative area change were significantly lower in patients with PH than those without PH (Table 2). Comparisons between subgroups of PH are shown in Table 3 and demonstrate that patients with severe pre-capillary PH such as IPAH and CTEPH have significantly worse RV function and mass than patients with PH owing to left heart disease.

**Table 2:** Demographics, RHC, Echocardiography and CMR Data

	**No PH n = 39**	**PH n = 194**
**Demographics**		
Age (yrs)	61 ± 16	63 ± 15
Female (%)	56%	59%
WHO class I		3 (1.5%)
II		42 (21.6%)
III		127 (65.5%)
IV		22 (11.3%)
**Invasive catheter measurements**		
mRAP (mmHg)	6 ± 3	11 ± 5†
mPAP (mmHg)	19 ± 3	45 ± 12†
PCWP (mmHg)	11 ± 5	12 ± 6
CO (L.min)	7.0 ± 1.8	5.1 ± 1.7†
CI (L.min.m^-2^)	3.7 ± 0.7	2.9 ± 0.9†
PVR (dyn.s.cm^-5^)	149 ± 167	587 ± 411†
Mixed VO2 (%)	73.5 ± 6.3	63.8 ± 9.2†
**Echocardiography (n = 195)**		
TRJV (cm/s)	2.6 ± 0.6	3.9 ± 0.9†
Echo derived mPAP (mmHg)	25.4 ± 7.6	46.8 ± 16.6†
**MR – RV morphology**		
RVEDVI (ml/m^2^)	77.7 ± 24.6	99.1 ± 39.0†
RV mass index (g/m^2^)	13.7 ± 6.92	37.6 ± 18.7†
VMI (ratio)	0.26 ± 0.11	0.70 ± 0.35†
Late gadolinium enhancement, ratio and % **(n = 159)**	2/31 (6%)	108/128 (86%)
**MR – RV function**		
RVEF (%)	39.7 ± 14.5	28.2 ± 14.5†
RVSVI (ml/m^2^)	31.4 ± 13.3	23.3 ± 13.2†
TAPSE (cm)	2.21 ± 0.66	1.55 ± 0.76†
f-TAAD (%)	24.6 ± 7.89	15.9 ± 7.49†
SFD (cm)	1.10 ± 0.45	0.64 ± 0.52†
f-SFD (%)	27.1 ± 11.5	14.4 ± 12.3†
RVRAC (%)	39.8 ± 10.5	26.2 ± 13.4†
sEI (ratio)	1.19 ± 0.19	1.57 ± 0.52†
dEI (ratio)	1.17 ± 0.12	1.28 ± 0.23
**Phase contrast – Pulmonary artery (n = 106)**		
Average velocity (cm/s)	13.6 ± 6.7	7.6 ± 3.4†
Retrograde flow (L/min/m^2^)	0.2 ± 0.1	0.5 ± 0.3†
Retrograde flow (%)	9.3 ± 7.2	16.3 ± 9.2†
PA relative area change ≥ 15%	17.8 ± 6.6	8.1 ± 6.5†
Systolic PA area (cm^2^)	7.8 ± 4.6	9.7 ± 2.8†
Diastolic PA area (cm^2^)	6.7 ± 4.7	8.9 ± 2.8†

Mean ± SD values displayed, † tested value significantly different in the PH patient group than in the ‘No PH’ group (*p* < 0.05).

**Table 3:** Subgroup analysis

	**Gp 1 IPAH n = 28**	**Gp 1 PAH-CTD n = 39**	**Gp 2 PH-LHD n = 21**	**Gp 3 RESP n = 29**	**Gp 4 CTEPH n = 59**
**Demographics and RHC**					
Age (yrs)	53 ± 17^¶, +, §, ƒ^	67 ± 9^#^	74 ± 9^#^	67 ± 12^#^	64 ± 14^#^
mPAP (mmHg)	54 ± 13^¶, +, §^	40 ± 13^#, ƒ^	35 ± 8^#, ƒ^	39 ± 8^#, ƒ^	49 ± 10^¶, +, §^
PCWP (mmHg)	11 ± 3^+^	11 ± 4^+^	21 ± 4^#, ¶, §, ƒ^	12 ± 4^+^	12 ± 5^+^
mRAP (mmHg)	11 ± 5	9 ± 5^+^	14 ± 5^¶, §^	9 ± 4^+^	12 ± 6
CO (L.min)	4.6 ± 1.4^+^	5.2 ± 1.6	6.2 ± 1.7^#, ƒ^	5.3 ± 1.3	4.8 ± 1.5^+^
CI (L.min.m-2)	2.5 ± 0.7^¶, +^	3.1 ± 0.7^#, ƒ^	3.4 ± 0.8^#, ƒ^	3.0 ± 0.6	2.5 ± 0.7^¶, +^
PVR (dyn.s.cm-5)	876 ± 439^¶, +, §^	484 ± 337^#, ƒ, +^	183 ± 87^¶, #, ƒ,^	428 ± 206^#, ƒ^	736 ± 373^¶, +, §^
**CMR**					
RVEDVI (ml/m^2^)	119 ± 47	82 ± 27^+, ƒ^	91 ± 38^¶^	96 ± 33	108 ± 41^¶^
VMI (ratio)	1.0 ± 0.4^¶, +, §^	0.6 ± 0.4^#, ƒ, +^	0.3 ± 0.1^#, ƒ, ¶^	0.6 ± 0.2^#, ƒ^	0.8 ± 3.1^¶, +, §^
RV mass index (g/m^2^)	50 ± 19^¶, +, §^	27 ± 14^#, ƒ^	22 ± 11^#, ƒ^	32 ± 11^#, ƒ^	43 ± 16^¶, +, §^
Late gadolinium enhancement ratio and %	21/22 (95%)^+^	20/28 (77%)	4/15 (27%)^#, ƒ^	19/20 (95%)	38/39 (97%)^+^
RVEF (%)	23 ± 15	32 ± 16 ^ƒ^	32 ± 10 ^ƒ^	24 ± 15	21 ± 12^¶, +^
RVSVI (ml/m^2^)	26 ± 21	24 ± 14	29 ± 13	22 ± 13	21 ± 11
TAPSE (cm)	1.5 ± 0.5	1.6 ± 0.7	2.0 ± 0.6^§, ƒ^	1.4 ± 0.7^+^	1.2 ± 0.5^+^
sEI (ratio)	2.2 ± 0.6^¶, +, §, ƒ^	1.4 ± 0.4^#^	1.2 ± 0.2^#, ƒ^	1.3 ± 0.3^#, ƒ^	1.6 ± 0.5^#,+, §^
dEI (ratio)	1.5 ± 0.4^¶, +, §, ƒ^	1.3 ± 0.2^#^	1.2 ± 0.2^#^	1.1 ± 0.1^#^	1.3 ± 0.3^#^
Average velocity (cm/s)	7.0 ± 3.0	10.0 ± 3.7 ^ƒ^	9.1 ± 3.4	7.4 ± 3.4	5.9 ± 2.5^¶^
Retrograde flow (L/min/m^2^)	0.5 ± 0.3	0.4 ± 0.3	0.5 ± 0.3	0.4 ± 0.2	0.5 ± 0.3
PA relative area change (%)	6 ± 5^+^	9 ± 6	13 ± 10^#, ƒ^	8.2 ± 6.0	6.5 ± 4.0^+^
Systolic PA area (cm^2^)	9.4 ± 1.8	9.0 ± 2.1	9.0 ± 2.2	9.1 ± 1.9	9.9 ± 2.2
Diastolic PA area (cm^2^)	8.9 ± 1.8	8.1 ± 2.1	7.8 ± 2.0	8.2 ± 2.0	9.2 ± 2.0

Mean ± standard deviation presented.

^#^: *P* < 0.05 in comparison to group 1, idiopathic pulmonary arterial hypertension (IPAH).

^¶:^*P* < 0.05 in comparison to group 2, pulmonary arterial hypertension associated with connective tissue disease (PAH-CTD).

^+^: *P* < 0.05 in comparison to group 3, pulmonary hypertension owing to left heart disease (PH-LHD).

^§^: *P* < 0.05 in comparison to group 4, pulmonary hypertension associated with lung disesae (PH-RESP).

^ƒ^: *P* < 0.05 in comparison to group 1, chronic thromboembolic pulmonary hypertension (CTEPH).

### Correlations with invasive haemodynamics

**Figure 3:**
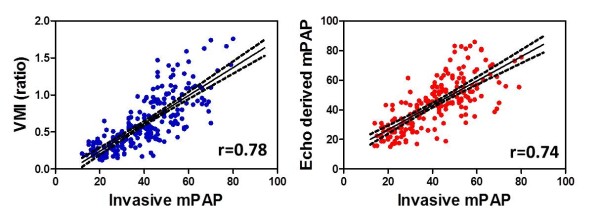
Scatter plots showing the significant linear relationships of VMI and Echocardiography derived mPAP shown on the y-axis versus right heart catheter measured mPAP on the x-axis.

Ventricular mass index demonstrated the strongest correlation of all quantitative MR measurements with mPAP (r = 0.78; *p* < 0.0001) and PVR (r = 0.74; *p* < 0.0001). Echo predicted mPAP correlated well with mPAP (r = 0.74; *p* < 0.0001), and with PVR (r = 0.74; *p* < 0.0001), Figure 3. sEI demonstrated a strong correlation with mPAP (r = 0.71; *p* < 0.0001) and PVR (r = 0.66; *p* < 0.0001), dEI demonstrated a significant though weaker correlations with mPAP, (r = 0.45; *p* < 0.0001) and PVR (r = 0.43; *p* < 0.0001). PA average velocity and PA relative area change demonstrated moderate negative linear correlations with mPAP r = −0.55, *p* < 0.0001 and r = −0.54, *p* <0.0001 respectively. Table 4 presents the correlations between all right ventricular morphological and functional MR indices with invasive haemodynamic metrics of mPAP and PVR.

**Table 4:** Correlations of CMR Parameters with mPAP and PVR

	**mPAP r value**	**p-value**	**PVR r value**	**p-value**
**Cardiac morphology**				
RVEDVI	0.39	<0.0001	0.42	<0.0001
RV mass index	0.74	<0.0001	0.65	<0.0001
VMI	0.78	<0.0001	0.74	<0.0001
**Right heart functional indices**				
RVEF	−0.43	<0.0001	−0.45	<0.0001
RVSVI	−0.24	0.001	−0.21	0.003
TAPSE	−0.40	<0.0001	−0.46	<0.0001
f-TAAD	−0.53	<0.0001	−0.50	<0.0001
SFD	−0.46	<0.0001	−0.40	<0.0001
f-SFD	−0.55	<0.0001	−0.42	<0.0001
RVRAC	−0.59	<0.0001	−0.56	<0.0001
sEI	0.71	<0.0001	0.66	<0.0001
dEI	0.45	<0.0001	0.43	<0.0001
**Phase contrast CMR**				
Average velocity (cm/s)	−0.55	<0.0001	−0.56	<0.0001
Retrograde flow (L/min/m^2^)	−0.33	<0.0001	0.25	0.029
Percentage retrograde flow (%)	−0.34	<0.0001	−0.31	0.001
PA relative area change (%)	−0.54	<0.0001	−0.54	<0.0001
Systolic PA area (cm^2^)	0.28	0.001	0.17	0.054
Diastolic PA area (cm^2^)	0.35	<0.0001	0.26	0.003

### Diagnostic accuracy

**Figure 4:**
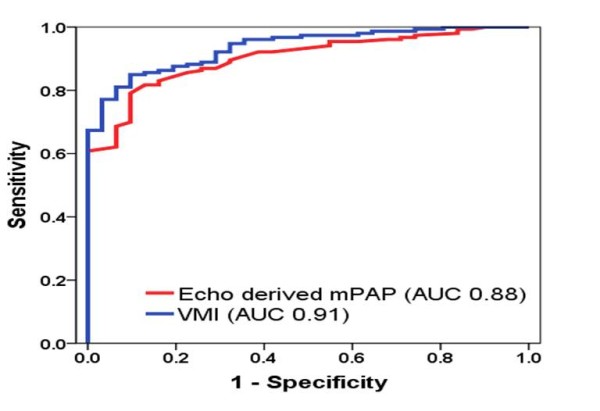
ROC curves showing the diagnostic accuracy of VMI and Echocardiography derived mPAP.

VMI and RV mass index were the CMR measurements with the highest diagnostic accuracy for the identification of PH from ROC curve analysis (AUC 0.91 for both), Figure 4. Identification of late gadolinium enhancement at the inter-ventricular hinge points was sensitive (83%) and specific (94%) for the identification of PH (AUC 0.89). Measures of right ventricular function were of only modest diagnostic accuracy for determining the presence of PH.

PA relative area change (AUC 0.87), PA retrograde flow (AUC 0.84), diastolic PA area (AUC 0.82) and average PA velocity (AUC 0.80) were the measurements made from phase contrast sequence images with the highest diagnostic accuracy for the detection of PH. PA relative area change < 15% was sensitive (86%) and specific (70%) for the detection of the presence of PH. PA retrograde flow greater than 0.3 L/min/m^2^ demonstrated sensitivity (82%) and specificity (71%) for the detection of pulmonary hypertension, more accurate than percentage retrograde flow with lower sensitivity (73%) and specificity (56%). PA area measured in systole (AUC 0.77) was a weaker marker of PH than diastolic PA area (AUC 0.82). Table 5 presents sensitivity, specificity, positive predictive value and negative predictive value and ROC analysis for the MRI measurements. Table 5 presents the CMR indices between subgroups of PH.

**Table 5:** Sensitivity, Specificity, Positive and Negative Predictive Values and area under the receiver operating characteristic curve (AUC) of CMR Indices for the Detection of PH

	**Sensitivity**	**Specificity**	**PPV**	**NPV**	**AUC**
**Cardiac morphology**					
RVEDV index ≥ 75 ml/m^2^	67	50	89	20	0.72^*^
RV mass index ≥ 20 g/m^2^	83	84	96	50	0.91^*^
VMI ≥ 0.4	81	88	97	50	0.91^*^
Late gadolinium enhancement	83	94	98	58	0.89^*^
**Right heart functional indices**					
RVEF ≤ 35%	67	71	93	28	0.72^*^
RVSVI ≤ 30 ml/m^2^	70	47	89	21	0.68^*^
TAPSE ≤ 2 cm	76	64	92	32	0.75^*^
f-TAAD ≤ 25%	88	56	92	44	0.78^*^
SFD ≤ 1 cm	77	67	93	34	0.73^*^
f-SFD ≤ 25%	79	64	92	35	0.78^*^
RVRAC ≤ 30%	61	81	95	28	0.79^*^
sEI ≥ 1.2	65	61	90	24	0.72^*^
dEI ≥ 1.17	62	54	88	20	0.57
**Phase contrast CMR**					
Average velocity ≤ 10 (cm/s)	82	62	94	32	0.80^*^
Retrograde flow ≥ 0.3 (L/min/m^2^)	83	71	95	38	0.84^*^
Retrograde flow (%)	73	56	87	34	0.75^*^
PA relative area change ≥ 15%	86	70	94	48	0.87^*^
Systolic PA area ≥ 8 cm^2^	74	67	92	32	0.77^*^
Diastolic PA area ≥ 6 cm^2^	88	66	94	52	0.82^*^

AUC (area under the curve) *p < 0.05.

**Figure 5:**
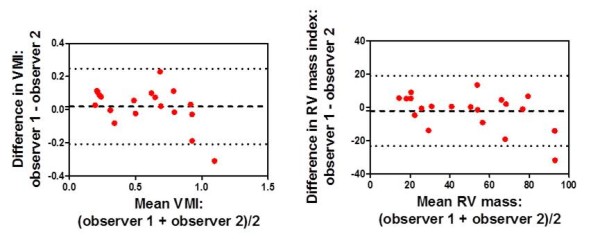
Bland Altman plots showing the agreement between two independent observers for ventricular mass index (VMI) and RV mass index measurements.

The bias between observers for CMR derived RV mass was −2.1 g (standard deviation 10.8) with limits of agreement −23.3 to 19.1 g. For VMI measurements the bias between observers was 0.02 (standard deviation 0.1) with limits of agreement of −0.21 to 0.24. See Figure 5**.**

The diagnostic accuracy of TRJV and echo derived mPAP had good diagnostic accuracy for predicting the presence of PH, AUC 0.86, and AUC 0.88 respectively,

## Discussion

This study has demonstrated that VMI and sEI were the CMR metrics with the strongest correlation with mPAP and PVR in the largest population of patients with suspected pulmonary hypertension studied to date undergoing CMR as part of their routine diagnostic work-up. VMI also demonstrated the highest diagnostic accuracy for the diagnosis of PH of all measured MR indices. In addition, late gadolinium enhancement at the inter-ventricular hinge points was sensitive and specific for the identification of PH and was present in 95% of patients with IPAH and 97% of patients with CTPEH, supporting a role for the routine measurement of these MR metrics in patients undergoing diagnostic CMR evaluation for suspected pulmonary hypertension.

CMR provides accurate and reproducible measurements of RV morphology and function, including mass, RVEDVI, RVEF, and RVSVI [25]. Currently manual RV volume and mass measurements are preferred to semi-automated methods with a manual technique showing stronger inter-observer agreement [7]. This study supports previous work assessing RVEDVI,,RV mass index and VMI which were all found to be elevated in PH when compared to patients with ‘No PH’. RVEF and RVSVI were significantly reduced compared to patients with ‘No PH’ [26,27]. TRJV and estimated mPAP measured at echocardiography had a good correlation with invasively measured mPAP and had high diagnostic accuracy for the detection of PH confirming that both echocardiography and CMR have diagnostic utility in patients with suspected PH, However, echocardiography and CMR may have limited value in an individual patient due for example to difficult acoustic windows or claustrophobia, respectively.

In the work-up of patients with suspected PH CMR may be particularly useful in patients in whom echocardiography is suboptimal, and this study identifies VMI and late gadolinium enhancement as the metrics of greatest diagnostic value. However, there is currently no reliable fully automated method for calculating RV mass, therefore RV muscle wall has to be manually segmented on each SA image slice. This is a time consuming process and is difficult to perform in patients with low RV mass as the wall is thin and is difficult to accurately measure, further work refining methods of determining RV mass are required [8,9]. RV mass intuitively increases over time due to elevated RV after load in patients with PH. We postulate that RV hypertrophy is an adaption mechanism of the RV in order to preserve function. The temporal relationship between RV mass and pulmonary artery pressure has not been fully described in the literature. The length of time an individual is exposed to high pulmonary artery pressure and their response to this increased after load will influence the degree of hypertrophy. Studying the temporal relationship between RV mass and mPAP would be of particular value when assessing RV responses to therapy.

Simple functional measurements such as left ventricular eccentricity indices have the advantage over other more labour intensive MR indices that they are easy and quick to measure, and can be performed in routine clinical practice whilst reviewing the SA images. Although these measurements have been investigated with echocardiography, there is little published evidence for their clinical utility using MRI. Given that the reproducibility of right ventricular measurements from echocardiography is accepted as challenging, validation of such measures with MRI is desirable. sEI assesses the effect of the ventricular pressure differential on the shape of the left ventricle, with values increasing as the septum bows further to the left side deforming the LV cavity and has been shown to correlate with pulmonary arterial and RV pressure using echocardiography in patients with PH [28]. Our MR derived sEI measurement correlated strongly with mPAP and PVR as shown with echocardiography [23,29,30]. We demonstrated similar results using MRI to those found using echocardiography with a sEI index >1.2 suggesting the presence of PH [23]. In patients with mild elevations in mPAP, the ventricular pressure gradient is not significant enough to cause paradoxical motion, this in part explains the poor sensitivity of sEI to detect the presence of PH and caution should be taken when interpreting sEI in patients with low mPAP.

Patients with PH have significantly more retrograde PA flow and significantly reduced average PA velocity than patients without PH. Retrograde PA flow >0.3 L/min/m^2^ and average velocity (10 cm/s) had reasonable diagnostic accuracy for the detection of PH in this studyl. Sanz et al. suggest that average velocity is the most robust phase contrast diagnostic measurement at the pulmonary artery [15], with reduced average velocity and increased pulmonary artery area occurring simultaneously as a response to increased pressure and resistance. However, retrograde flow outperformed average velocity as a diagnostic tool in our study, and our finding supports a recent study showing that early retrograde flow is a characteristic feature in patients with PH [31].

We have also summarised the differences in haemodynamic and CMR parameters between the larger sub-groups of patients in this study, Table 3. Patients with IPAH and CTEPH had evidence of severe pulmonary hypertension at right heart catheter and 95% and 97% of patients respectively had delayed myocardial enhancement demonstrating that this metric is highly sensitive for the detection of severe increases in RV after load seen IPAH and CTEPH. Importantly qualitative assessment of late gadolinium enhancement can be performed rapidly. Patients with IPAH and CTEPH had more severe disease at cardiac catheter than patients with SSc-PAH and this is reflected in CMR morphological findings with increased RV mass index and increased VMI and more severe functional impairment than in IPAH and CTEPH compared to PAH-CTD. There is little published data of CMR RV findings in patients with LV diastolic dysfunction. Importantly this study demonstrates that VMI is low in these patients and they have much better preserved cardiac function than patients with pre-capillary disease such as IPAH and CTEPH where pulmonary vascular resistance is significantly elevated. This is likely to reflect the site of disease with patients with post-capillary disease such as LHD only having minimally elevated pulmonary vascular resistance despite high pressures. The low prevalence of late gadolinium enhancement in 27% in patients with PH-LHD is consistent the primarily post-capillary nature of pulmonary vascular disease in the majority of these patients. Patents with PH-RESP had moderate elevation of mPAP and PVR and elevated VMI and impaired RV function consistent with a significant increase in RV after load.

### Limitations

The MRI measurements were acquired retrospectively. Echocardiography was performed several weeks prior to admission for RHC, this limits the direct comparison of the diagnostic accuracy of CMR versus echocardiography for the detection of PH. Echocardiography is a well-established screening tool in the evaluation of patients with suspected PH, implementation of CMR as a replacement for echocardiography would be associated with several challenges, including cost, availability and expertise. The control or “No PH” group includes patients referred with suspected PH due to clinical signs/symptoms or an abnormal echocardiogram suggestive of PH, but were found to have a normal pulmonary artery pressure at RHC. These patients frequently had co-morbidities and cannot be considered to be healthy controls. This importantly, however, represents the target population that one would consider performing such diagnostic tests and prevents some of the errors inherent in analysing data exclusively from particular sub-groups. Several patients in the ‘No PH’ group, however, had right ventricular dysfunction on CINE cardiac MRI scans in the absence of significant pulmonary vascular disease which may have contributed to the reduced accuracy of MR indices.

## Conclusions

MRI is a robust alternative to echocardiography in the evaluation of patients with suspected PH. VMI showed a linear relationship with mPAP and had the highest accuracy of all CMR measurements for the identification of PH. Late gadolinium enhancement, PA relative area change and retrograde flow are other useful markers that should be evaluated in patients with suspected pulmonary hypertension who undergo CMR.

## Abbreviations

dEI, Diastolic eccentricity index; PA, Pulmonary artery; PH, Pulmonary hypertension; RVEDVI, Right ventricular end-diastolic volume index; RVEF, Right ventricular ejection fraction; RVSVI, Right ventricular stroke volume index; sEI, Systolic eccentricity index; TRJV, Tricuspid regurgitant jet velocity; VMI, Ventricular mass index.

## Competing interests

SR is funded by an unrestricted educational grant from Pfizer. DC funded by Bayer. JH is part funded as a clinical research fellow by an unrestricted educational grant from Actelion and has received conference funding from Actelion, GSK and Pfizer. CD has received conference funding from Pfizer. CH has received conference funding from Pfizer. CAE has sat on advisory boards for, received consultancy and lecturing fees from and been funded to attend conferences from Actelion, Bayer, Encysive, GSK, Pfizer and United Therapeutics. RC has received honoraria for lecturing from Actelion and GSK, has sat on advisory boards for Actelion and Bayer and has received conference funding from Actelion, GSK, Pfizer and United Therapeutics JMW has received unrestricted educational grants from GE and GSK and conference funding from Bayer. DGK has sat on advisory boards for, received consultancy and lecturing fees from and been funded to attend conferences from Actelion, Bayer, Encysive, GSK, Pfizer, Lilley and United Therapeutics.

## Authors' contributions

AS and DGK conceived the idea for the study. AS, SR, JH, RC, CAE, JW and DGK participated in the study design. AS, SR, DC acquired the MRI data. Image analysis was performed by AS and SR. AS, RC, JMW, DGK analysed and interpreted the MR data. AS, SR, JMW and DGK drafted the manuscript. All authors read and approved the final manuscript**.**
